# Frailty Risk Model for Readmission after Transoral Robotic Surgery for Oropharyngeal Squamous Cell Carcinoma

**DOI:** 10.51894/001c.164370

**Published:** 2026-07-31

**Authors:** Hussein Saleh, Izza Ali, Katrina M. Minutello, Abdul Nashwati, Jonah Zeitlin, Hassan B. Nasser

**Affiliations:** 1 Henry Ford Wyandotte Hospital

## 37

### Background

Accurate preoperative risk stratification is essential in patients undergoing transoral robotic surgery (TORS) for oropharyngeal squamous cell carcinoma (OPSCC), yet the impact of frailty on short-term outcomes following transoral robotic surgery (TORS) remains poorly characterized.

### Objectives

To determine whether preoperative frailty is associated with increased risk of 30-day readmission, prolonged hospital length of stay, non-home discharge, reoperation, and mortality.

### Methods

A retrospective cross-sectional analysis used the American College of Surgeons National Surgical Quality Improvement Program (ACS-NSQIP) database from 2022 to 2024. All adult patients undergoing TORS for OPSCC were identified using International Classification of Diseases codes and included. Exclusion criteria were: age younger than 18 years, incomplete information regarding frailty index factors, or readmission.

### Results

Among 378 patients undergoing TORS for OPSCC, 43 (11%) were classified as frail by mFI-5 score. Frail patients were significantly older, had higher body mass index (BMI), and were more likely to have American Society of Anesthesiologists (ASA) physical status classification 3-4 disease. According to multivariable analysis, frailty was independently associated with increased odds of 30 day unplanned reoperation (OR 3.30, 95% CI 1.08-9.87). Frail patients also had longer median hospital stays (3 vs. 2 days) and higher rates of non-home discharge (12% vs. 0%). Operative times and concurrent neck dissections were independent predictors of prolonged length of stay.

### Conclusion

Frailty measured by mFI-5 is independently associated with increased risk of reoperation following TORS for OPSCC. Routine frailty screening may be helpful in identifying patients warranting closer perioperative attention as well as a more thorough preoperative counseling regarding surgical risk.

